# The Fast Changing Landscape of Sequencing Technologies and Their Impact on Microbial Genome Assemblies and Annotation

**DOI:** 10.1371/journal.pone.0048837

**Published:** 2012-12-12

**Authors:** Konstantinos Mavromatis, Miriam L. Land, Thomas S. Brettin, Daniel J. Quest, Alex Copeland, Alicia Clum, Lynne Goodwin, Tanja Woyke, Alla Lapidus, Hans Peter Klenk, Robert W. Cottingham, Nikos C. Kyrpides

**Affiliations:** 1 DOE Joint Genome Institute, Walnut Creek, California, United States of America; 2 Oak Ridge National Laboratory, Oak Ridge, Tennessee, United States of America; 3 Los Alamos National Laboratory, Bioscience Division, Los Alamos, New Mexico, United States of America; 4 Leibniz Institute DSMZ - German Collection of Microorganisms and Cell Cultures, Braunschweig, Germany; Auburn University, United States of America

## Abstract

**Background:**

The emergence of next generation sequencing (NGS) has provided the means for rapid and high throughput sequencing and data generation at low cost, while concomitantly creating a new set of challenges. The number of available assembled microbial genomes continues to grow rapidly and their quality reflects the quality of the sequencing technology used, but also of the analysis software employed for assembly and annotation.

**Methodology/Principal Findings:**

In this work, we have explored the quality of the microbial draft genomes across various sequencing technologies. We have compared the draft and finished assemblies of 133 microbial genomes sequenced at the Department of Energy-Joint Genome Institute and finished at the Los Alamos National Laboratory using a variety of combinations of sequencing technologies, reflecting the transition of the institute from Sanger-based sequencing platforms to NGS platforms. The quality of the public assemblies and of the associated gene annotations was evaluated using various metrics. Results obtained with the different sequencing technologies, as well as their effects on downstream processes, were analyzed. Our results demonstrate that the Illumina HiSeq 2000 sequencing system, the primary sequencing technology currently used for de novo genome sequencing and assembly at JGI, has various advantages in terms of total sequence throughput and cost, but it also introduces challenges for the downstream analyses. In all cases assembly results although on average are of high quality, need to be viewed critically and consider sources of errors in them prior to analysis.

**Conclusion:**

These data follow the evolution of microbial sequencing and downstream processing at the JGI from draft genome sequences with large gaps corresponding to missing genes of significant biological role to assemblies with multiple small gaps (Illumina) and finally to assemblies that generate almost complete genomes (Illumina+PacBio).

## Introduction

Prior to 2004, nearly all DNA sequencing used the chain-termination method developed by F. Sanger [1]. Typically a Sanger sequencing machine yields about 1.5 Mbp/day of high-quality reads with an average length of 500–800 bases. However, the fragments of DNA to be sequenced must first be cloned and the resulting libraries maintained. Next generation sequencing (NGS) technologies bypass cloning by immobilizing the DNA fragments and subjecting them to sequential interrogations. Widely used technologies, such as 454 pyrosequencing [2] and Illumina sequencing-by-synthesis [3], use DNA polymerase to drive their sequencing reactions but do not require cloning, Pacific Biosciences use a sequencing by synthesis technology which is applied on single molecule in real time [4]. Illumina produces reads which are now routinely 150 bases in length and can be extended up to 250 bases using overlapping paired end reads; output is ∼60 Gb per lane or 420 Gb per flowcell. Read length for the 454 platform now exceeds 600 bases; output is 10 Gb per run.

Their low cost, simplicity of library generation and instrument operation, and quantity of data generated have made the NGS technologies, alone or in combination, an attractive choice for microbial genome sequencing projects. The quality of the generated sequence is, on many occasions, lower than the Sanger standards, but the high coverage obtained allows for the correction of sequencing errors. However, the shorter read length still makes assembly challenging. Regardless of the specific NGS technology used, the result of the first pass assembly represents a *draft* version for the majority of the genomes that comprises many contigs, some of which are incorrectly assembled, and also presumably contains sequencing errors. Currently the quality of the draft genome (assessed as the number of contigs generated) is a function not only of the quality of the machine-generated read sequences but also of the proficiency and limitations of the downstream processes (assembly and annotation) and algorithms used.

The *finished* or *noncontiguous finished* versions according to Chain et al [5] of the genome are high quality assemblies that have been manually checked and improved, with all gaps closed or filled and misassemblies corrected so that each replicon appears as a single contiguous sequence. The generation of such high-quality data is costly, necessitates special skills, and requires time-consuming manual work. Considering the current genome finishing rate versus the number of sequenced genomes per year, finishing each sequenced genome is not feasible. As a result, an increasingly large number of sequenced genomes remain unfinished, at a “permanent draft” stage, which is used for subsequent analyses. Before proceeding with such analyses, it is essential to evaluate the consensus error rate and correctness of those assemblies. Furthermore, given the numerous sequencing technologies now in use, it is critical to know the capabilities and limitations of each, and to design and evaluate sequencing projects on this basis.

Here we present an evaluation of current sequencing technologies based on analysis of 133 microbial genomes sequenced during the last seven years at the Department of Energy-Joint Genome Institute (DOE-JGI). We use these data to evaluate the quality of the assembled product and, in particular, to compare the draft products resulting from automated assemblies with the finished genomes.

## Results and Discussion

### Genomes and technologies surveyed

**Figure 1:**
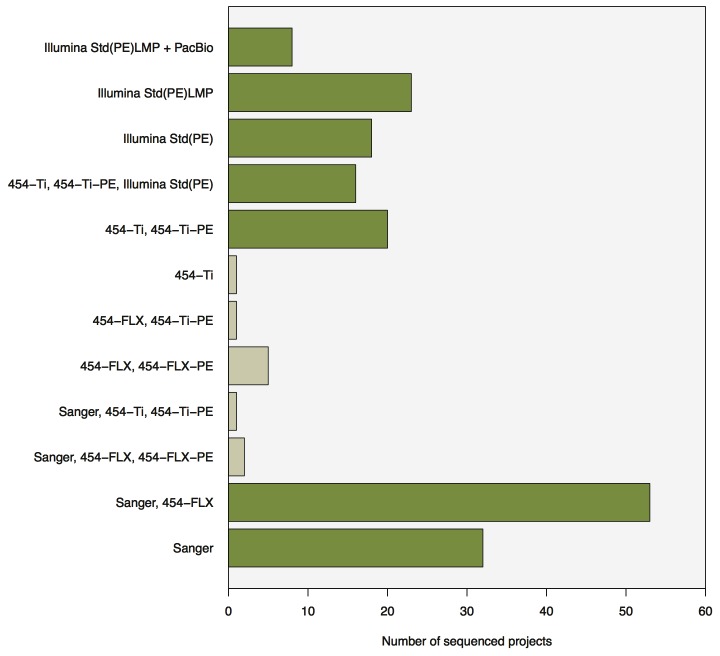
The distribution of projects among the 12 sequencing methods used. With dark green color are indicated the projects for which there are more than 5 sequenced projects and were used in downstream analysis.

During the last 7 years, 133 microbial genomes were sequenced to completion at the DOE-JGI (Table S1). These sequencing projects were carried out using a variety of sequencing technologies, alone or in combination (Table 1 and Figure 1). Several projects specifically compared different variants of a method (e.g., Illumina vs Illumina+PacBio). Included are draft and finished genomes that were submitted to Genbank and that included only contigs that were >200 bp. This size threshold was used in compliance with NCBI rules for submission of data from sequencing projects. The projects selected span the full spectrum of the GC percentage and phylogenetic placement (Table S1). These projects were sequenced until the end of 2011, however the current technology and methods used are undergoing constant improvements, which result in significant better results e.g. Illumina transitioned from V2 to V3 chemistry with significant improvement in the final product. Additionally improvements in the software used to process these data have been reflected in the quality of the end product as well. The purpose of this report is not to thoroughly evaluate these differences but is focused on the differences observed while transitioning from one technology to another, and the resulting quality of the assembled and annotated product.

**Table 1:** Methods used in this comparison.

Method name	Description
Sanger	Standard sequencing using the Sanger method. Results in long reads of average size >500 bp.
Sanger, 454 – FLX	Previous sequencing technology with additional reads from 454-FLX chemistry. 454-FLX were reads of average size >200 bp.
Sanger, 454 –FLX, 454-FLX-PE^1^	Previous sequencing technology with additional paired end reads from 2–20 kbp 454 libraries.
Sanger, 454-Ti, 454-Ti-PE^1^	Standard sequencing using the Sanger method with additional reads from 454-Ti chemistry. 454-Ti were reads of average size >450 bp. Paired reads were from libraries of 2–20 kbp insert size.
454-FLX, 454-FLX-PE^1^	454-FLX chemistry with additional paired end reads from libraries of 2–20 kbp insert size.
454-FLX, 454-Ti-PE^1^	454-FLX chemistry with additional paired end reads from libraries of 2–20 kbp insert size sequenced with 454-Ti chemistry.
454-Ti	Sequence reads using single 454-Ti chemistry.
454-Ti, 454-Ti-PE^1^	Previous technology with additional paired end reads from libraries of 2–20 kbp insert size sequenced with 454-Ti chemistry.
454-Ti, 454-Ti-PE^1^, Illumina Std(PE^1^)	Previous technology with additional paired end reads from libraries of 200–300 bp insert size sequenced with the Illumina Genome Analyzer IIx. Reads from Illumina had a length of 75,100 and 150 bp.
Illumina Std(PE^1^)	Sequencing was performed using only Illumina reads with paired end reads from libraries of 200–300 bp insert size.
Illumina Std(PE^1^) LMP^2^	Previous sequencing technology with additional paired end reads from long mate pair libraries up to 18 kbp insert size.
Illumina Std(PE^1^)LMP^2^, PacBio	Previous sequencing technology with additional reads from PacBio DNA sequencing system. PacBio results in reads of average size ∼500 bp with reads potentially up to several kb.

1 PE: paired end reads.

2 LMP: Long Mate Paired reads.

### Quality of assembly

Two metrics were used to evaluate the quality of the produced assembly: the number of contigs in the draft assembly and the amount of missing DNA sequence, i.e., number of bases in the finished assembly that is not included in the draft. In both cases higher numbers indicate worse quality of assembly resulting in loss of information about the genome e.g. missing genes, gene context information, and make downstream analysis more difficult.

**Figure 2:**
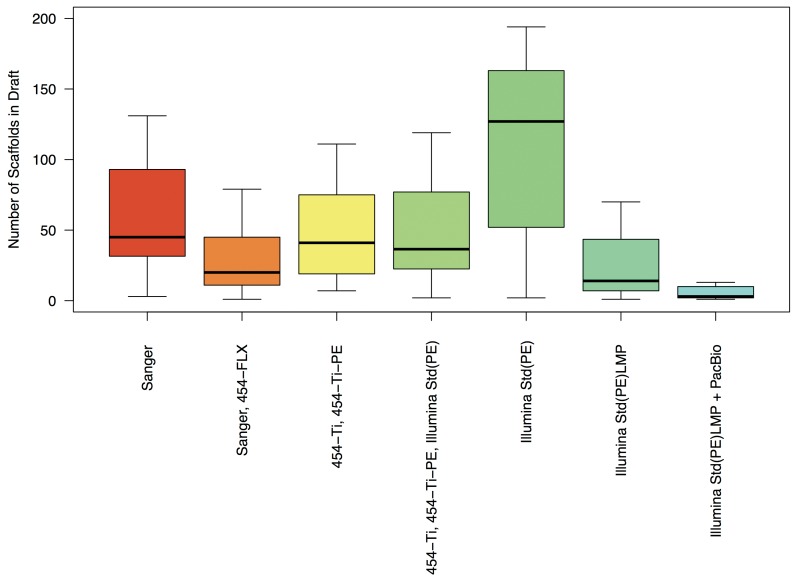
Assembly quality as assessed by the number of scaffolds in draft assemblies. Data is shown for the six sequencing methods with more than 5 projects. Indicated are the range from upper to lower quartile (boxes), the median (thick black line), and the minimum/maximum values.

Overall NGS technologies yield fewer contigs compared to Sanger-based sequencing (Figure 2). The 454 technology alone produces better results than Sanger alone; combining Sanger with 454 reduces the number of scaffolds further. In comparison, standard Illumina yields more draft scaffolds, but the number is significantly reduced when long mate pair libraries are used or when Illumina is combined with 454, and more so when combined with PacBio sequence data.

**Figure 3:**
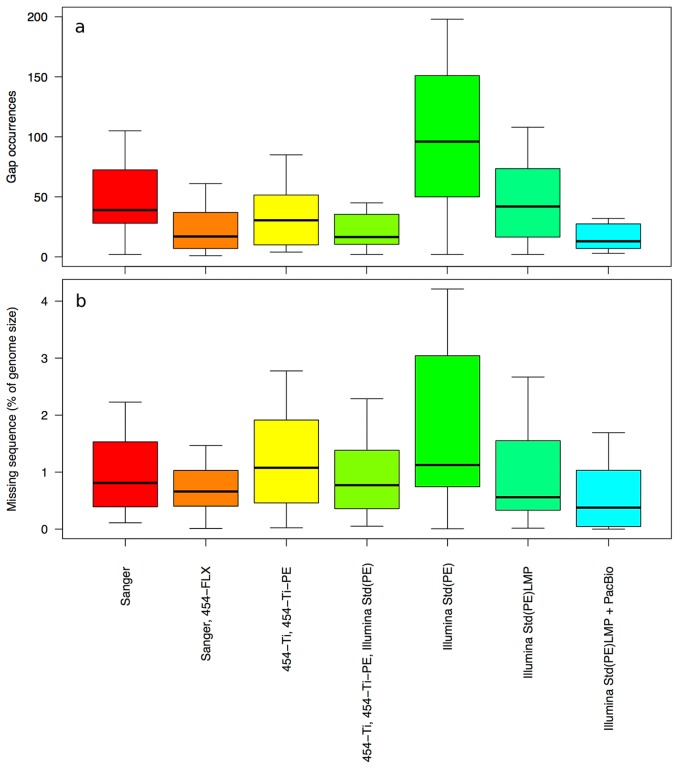
Assembly quality for the draft genomes included in this analysis. Assembly quality is assessed by (a) the number of gaps in the draft assemblies, and (b) gap size expressed as a percentage of genome length. Data is shown for the six sequencing methods with more than 5 projects.

Each region of the finished genome that is missing from the draft assembly was identified as a gap. The number of gaps (gap occurrences) per genome (Figure 3A) and their total size expressed as the percentage of the genome length (Figure 3B) were compared for seven combinations of technologies. Generally the NGS technologies yield fewer gaps, with Illumina-based technologies being the exception. Conversely, Illumina-based methods produce shorter gaps than Sanger alone, while 454-based methods yield longer gaps. Including paired end libraries in the case of Illumina-based assemblies improves the measured assembly metrics. Notably, sequenced reads generated by either Illumina or 454 sequencing technology typically cover the entire genome sequence (with the exception of very extreme GC% regions) [6]–[7]. Thus, the observed gaps in the draft assemblies are not sequencing gaps, but rather the result of weaknesses of the assembly algorithms and/or the exclusion of very short contigs (<200 bp) from the genomes included in this analysis.

**Figure 4:**
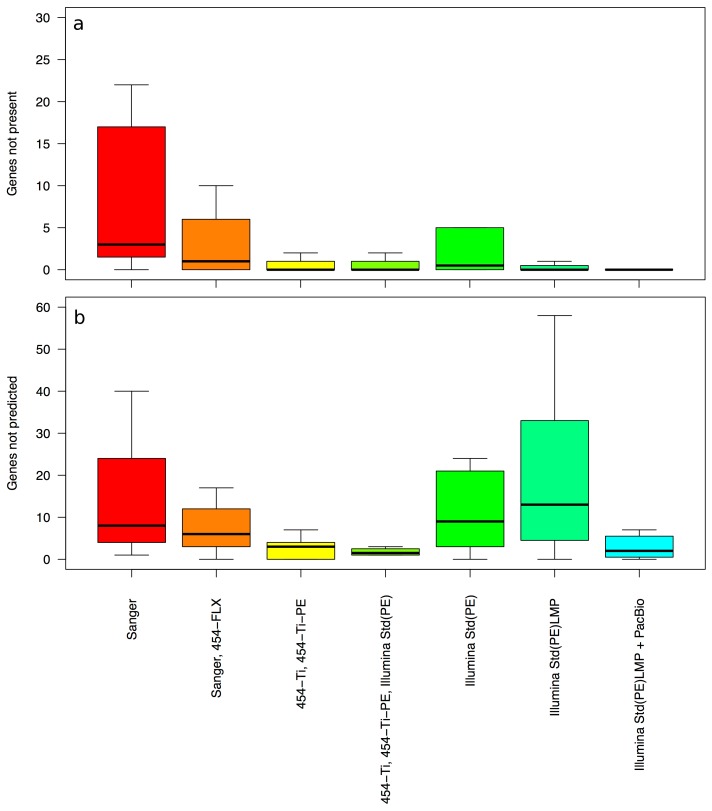
Genes missed in draft assemblies. Data is shown for the sequencing methods with more than 5 projects. (a) Missed gene sequences, i.e., the number of genes in the finished genome whose nucleotide sequence is absent from the draft assembly. (b) Unrecognized genes, i.e., the number of genes whose nucleotide sequence is present in the draft assembly but that were not predicted by Prodigal (v2.5).

The sequences missing from the draft assemblies were also evaluated in terms of the number of gene sequences missed. Direct comparison of base sequences showed that the number of *missed gene sequences* is low in most cases when the original sequencing employed NGS technologies (Figure 4A). In particular, when Illumina is used, this number averages close to zero, despite the putative misassemblies and assembly gaps. However, when comparing to the actual genes predicted on the draft genomes by *ab initio* gene predictors such as Prodigal [8] or GeneMark [9], the number of *unrecognized genes* is higher. In this case, part of the DNA sequence that codes for the gene is present in the assembled draft genome, but the gene prediction algorithms fail to identify it. The number of missing genes in Illumina-based assemblies is similar to that for Sanger-based assemblies (Figure 4B). Closer inspection revealed that the greater number of genes unrecognized with the *ab initio* gene predictors was due to the extend of fragmentation in the draft genome. The larger number of contigs resulted in many fragmented genes, frequently at the ends of contigs, which the gene callers typically miss. Better assemblies combined with similarity-based corrections (GenePRIMP [10]) can alleviate that and fill in these missing genes.

**Figure 5:**
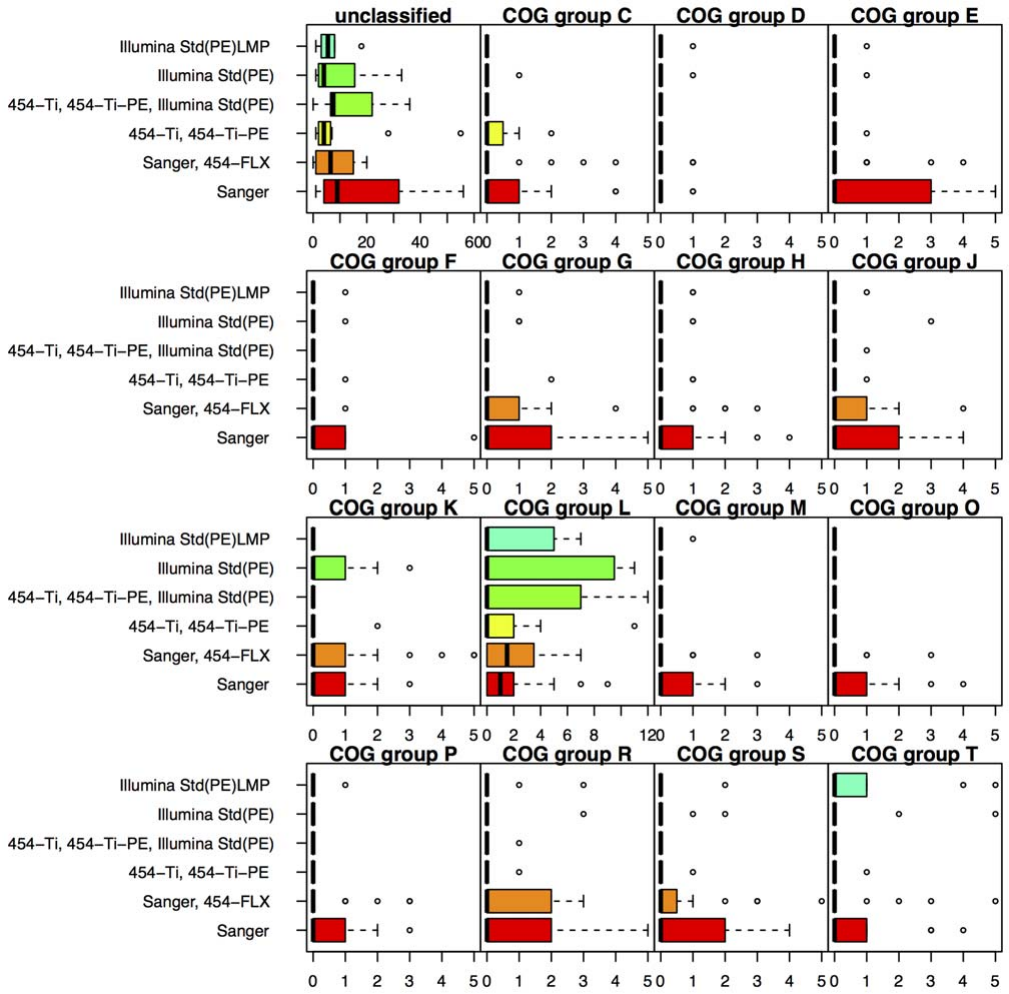
Misassemblies as detected by low gene quality. Low quality genes are genes present in the finished genome that had a similarity (tBLASTn) to the draft genome but the alignment was either short (<50% of the gene length) or identity was <90%. Data is shown for the six sequencing methods with more than 5 projects.

When the missed gene sequences were categorized based on their annotated COG function, their distribution was found to differ for the various sequencing technologies (Figure 5). For the projects sequenced by Sanger alone, they are distributed over many different COG groups. Among those previously found [11] to often be missing from Sanger-based sequences are ribosomal proteins (COG group J) and DNA polymerases (COG group L). In contrast, when using any of the NGS technologies, the missed gene sequences tend to be from only one or two groups, most often COG group L. This group includes transposases and related proteins, often present as multi-copy genes that form repeats that the assemblers cannot resolve. In all cases though the median number of missing genes is low.

### Misassemblies

To detect misassemblies, we compared the protein sequences of predicted genes between the draft and finished versions of each genome. The finished version served as the standard. Draft gene sequences that represented fragments or had low similarity to the finished sequence were assumed to be located in genomic regions that were misassembled. This metric does not directly measure the fidelity of the assembly method (i.e., the generation of misassemblies) however, it reflects the quality of the assembled sequence used for annotation and thus can be used as a proxy for assembly fidelity.

**Figure 6:**
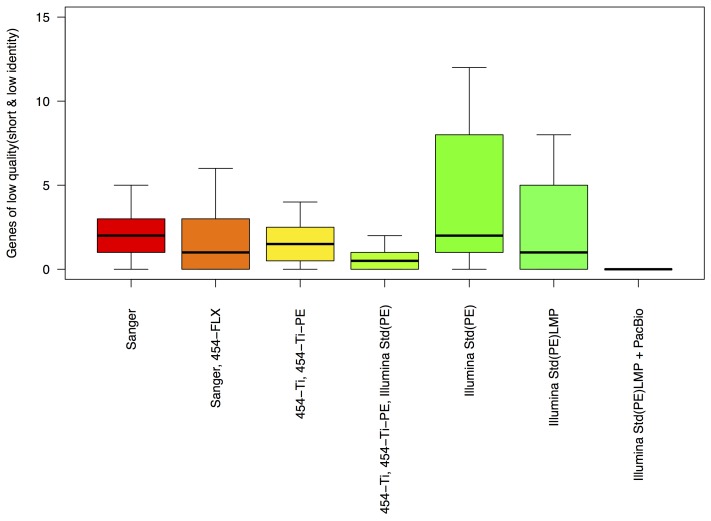
Distributions of functions, based on COG group assignments, of gene sequences missing in draft assemblies. Data is shown for six sequencing technologies; omitted is Illumina PacBio for which there are currently only eight genome projects without any missing genes.

Notably, assembly of reads generated by Illumina alone yielded more gene discrepancies (Figure 6), indicating that the assembled sequence contains either misassemblies (resulting in genes with low identity and truncated genes) or short contigs that contain gene fragments (resulting in truncated genes). To address this issue, short genes located at the end of draft contigs were excluded from these analyses.

### Effect of genome properties on assembly

The effect of three genome properties (GC%, number of repeats and genome size) on the quality of assembly was investigated using the number of draft contigs as a proxy for assembly quality (Table 2). Unexpectedly, the number of draft contigs shows no correlation with genome GC%. This can be attributed to the use of public draft assemblies in the analysis which often included multiple libraries or alternate chemistries to compensate for the poor quality of the initial assembly due to GC biases.

**Table 2:** Correlation of the number of contigs with genome GC%, repeat content, and size.

Technology	GC %	Short repeats	Medium repeats	Long repeats	Genome size
Sanger	0.091	0.356 *	0.277 *	0.170	0.356 *
Sanger, 454-FLX	0.017	0.372 *	0.355 *	0.224 *	0.278 *
454-Ti, 454-Ti-PE	0.032	0.525 *	0.721 *	0.579 *	0.249
454-Ti, 454-Ti-PE, Illumina Std(PE)	0.168	0.276	0.295	0.295	0.360
Illumina Std(PE)	0.255	0.373 *	0.342	0.135	0.556 *
Illumina Std(PE)LMP(I)	0.047	0.647 *	0.44 *	0.481 *	0.485 *
Illumina Std(PE)LMP(II)	−0.370	0.540	0.89 *	0.167	0.077
Illumina Std(PE)LMP+PacBio	−0.118	0.749 *	0.526	0.341	0.355

Data shown are the Kendall rank correlation coefficients.

* = pvalue<0.05.

It is known that a large number of repeats poses a problem during assembly, especially when the repeats are longer than the reads or inserts used [12]–[14]. As expected a correlation between the repeat content and the number of contigs was observed here, mostly with NGS-based sequencing, although weaker than expected. Similarly, there was only a weak correlation between genome size and the number of contigs. Here, too, the absence of bias in the public draft assemblies reflects the implementation of compensatory steps taken during sequencing or analysis.

## Conclusions

Our analyses show that the use of Illumina-based sequencing technologies for microbial genome projects is not only cost effective but can generate the entire sequence without significant loss of information, similarly to what other studies have shown [15]. Even when the genome is fragmented into multiple scaffolds, the amount of missing sequence is minimal, thus very few genes are actually missed. Furthermore, these sequencing technologies are free of the biases inherent in Sanger sequencing that resulted in the omission of housekeeping genes (e.g., DNA polymerase and ribosomal proteins). However, due to the short length of reads and of the paired end reads generated, assembly frequently yields a genome that is fragmented into many contigs and missing or misassembled repeat regions [16]. As a result, annotation methods have problems predicting some genes, particularly those located at the ends of contigs.

Finishing is an important step in the genome sequencing process that can provide high quality data, but it is costly and time-consuming. The analyses reported here indicate that, with the continuing improvement of assembly and annotation methods, draft sequences could be adequate for many purposes and finishing could be reserved for special situations. It is also providing evidence that the quality of the draft microbial genomes in the era of NGS sequencing technologies, are significantly better from the draft genomes of the sanger era, in terms of missed genes. Cutting-edge sequencing technologies, particularly in complementary combinations, provide a route to further improvement in assemblies and the quality of the predicted genes. Initial evidence, based on only four genomes, suggests that Illumina plus PacBio may yield higher quality results. We anticipate that the upcoming improvements of these technologies alone or in combination with the 3^rd^ generation sequencing technologies, will provide us with completely (or very close to) finished genomes, and will convert the laborious, costly and time consuming step of finishing, eventually obsolete.

## Methods

### Mapping of draft contigs to a finished genome

Comparisons between the finished and draft versions of each genome were performed using the NUCmer pipeline (part of MUMmer [17]) with no options, using the finished sequence as the ‘reference’ and the draft sequence as the ‘query.’ The alignments were mapped to the finished genome and each aligned base position designated as ‘mapped.’ These alignments provided the number of covered bases in the finished genome and the locations of gaps, i.e., regions missing from the draft contigs.

### Characterization of gaps

To characterize the content missing in the draft contigs, Prodigal [8] (v2.5) was used to predict protein coding genes on the draft contigs. Proteins encoded in the finished genome were then compared with those predicted in the draft genome using NCBI BLASTp [18]. Each protein in the finished genome was assigned to one of the following groups: identical proteins in both versions; similar full- length proteins (e.g., a sequence correction); longer in the draft and 100% identical (e.g., likely a frameshift); low quality hits (e.g., probably not in the draft), and proteins that had no hit.

To determine if the missing protein coding genes (belonging to the last two groups) were actually present in the draft sequence but had not been predicted by Prodigal, tBLASTn was used to search for those genes in the draft contigs.

### Identification of repeats

A repeat content ‘profile’ was generated for each genome that included both the repeat lengths (bp) and the number of occurrences for each. Megablast was run on each genome against itself. Then the RECON tool [19] was used to group the repeats into families and to screen for repeats that are at least 50 bases long and 95% identical to each other.

## Supporting Information

Table S1 **List of genomes and their features used for this study.**
(XLS)
